# Comprehensive Transcriptome Profiling of NAFLD- and NASH-Induced Skeletal Muscle Dysfunction

**DOI:** 10.3389/fendo.2022.851520

**Published:** 2022-02-21

**Authors:** Mingwei Guo, Liping Xiang, Jing Yao, Jun Zhang, Shuangshuang Zhu, Dongmei Wang, Caizhi Liu, Guoqiang Li, Jiawen Wang, Yuqing Gao, Cen Xie, Xinran Ma, Lingyan Xu, Jian Zhou

**Affiliations:** ^1^ Shanghai Key Laboratory of Regulatory Biology, Institute of Biomedical Sciences and School of Life Sciences, East China Normal University, Shanghai, China; ^2^ Department of Endocrinology and Metabolism, Shanghai Clinical Center for Diabetes, Shanghai Diabetes Institute, Shanghai Jiao Tong University Affiliated Sixth People’s Hospital, Shanghai, China; ^3^ State Key Laboratory of Drug Research, Shanghai Institute of Materia Medica, Chinese Academy of Sciences, Shanghai, China

**Keywords:** NAFLD, NASH, quadriceps muscle, lipid deposition, insulin resistance, myokines

## Abstract

Nonalcoholic fatty liver disease (NAFLD), characterized by extensive triglyceride accumulation in hepatocytes, may progress to nonalcoholic steatohepatitis (NASH) with liver fibrosis and inflammation and increase the risk of cirrhosis, cancer, and death. It has been reported that physical exercise is effective in ameliorating NAFLD and NASH, while skeletal muscle dysfunctions, including lipid deposition and weakness, are accompanied with NAFLD and NASH. However, the molecular characteristics and alterations in skeletal muscle in the progress of NAFLD and NASH remain unclear. In the present study, we provide a comprehensive analysis on the similarity and heterogeneity of quadriceps muscle in NAFLD and NASH mice models by RNA sequencing. Importantly, Gene Ontology and Kyoto Encyclopedia of Genes and Genomes pathway functional enrichment analysis revealed that NAFLD and NASH led to impaired glucose and lipid metabolism and deteriorated functionality in skeletal muscle. Besides this, we identified that myokines possibly mediate the crosstalk between muscles and other metabolic organs in pathological conditions. Overall, our analysis revealed a comprehensive understanding of the molecular signature of skeletal muscles in NAFLD and NASH, thus providing a basis for physical exercise as an intervention against liver diseases.

## Introduction

Nonalcoholic fatty liver disease (NAFLD) and nonalcoholic steatohepatitis (NASH) are common and complex chronic liver diseases, usually accompanied with systematic metabolic dysfunctions including obesity, diabetes mellitus, and sarcopenia, which increase the risks of cirrhosis, cancer, and death (1). Clinically, NAFLD is mainly manifested as liver steatosis with an accumulation of fat droplets in hepatocytes, while chronic NALFD may lead to NASH, characterized by hepatocyte fibrosis and inflammation, in addition to severe steatosis (2). However, effective drugs treating NAFLD and NASH are still lacking (3). Notably, lifestyle interventions, including physical exercises, are the primary recommendations for patients with NAFLD or NASH (4, 5). Indeed patients with NAFLD and NASH have been shown to exhibit impairments in skeletal muscle structure and functionality as well as an increased risk of cardiovascular diseases (6, 7). In addition, increasing evidence suggests that NAFLD and NASH are independent risk factors for muscle wasting and reduced muscle mass and strength (8, 9), while the detailed molecular changes of skeletal muscle in NAFLD and NASH are still unclear.

The skeletal muscle is a highly dynamic tissue that undergoes continuous remodeling in response to physiological and pathological conditions and is vital for energy homeostasis (10). Skeletal muscle fiber types are classified as oxidative (slow twitch) and glycolytic (fast twitch) fibers following the criteria of histochemical assessment, speed of twitch contraction, fatigability, and myosin heavy chain isoform expression (11). Impaired functionality and metabolic disturbance in skeletal muscles are key features and even the driving forces for obesity, NAFLD, NASH, and type 2 diabetes, which is termed as “sarcopenic obesity” (12), suggesting the importance of studying the molecular signature of the skeletal muscle and the crosstalk mechanisms during metabolic diseases (10).

Indeed the skeletal muscle exhibits great impacts on systematic energy homeostasis and communicates with central systems and peripheral metabolic tissues *via* specific myokines (13, 14)—for example, interleukin-6 has been reported to mediate glucose homeostasis in the liver during skeletal muscle contraction (15). Meteorin-like (*Metrnl*) serves as a critical regulator of muscle regeneration and is associated with reduced immune cell infiltration (16). Besides this, irisin, a myokine cleaved from membrane protein FNDC5, has been shown to drive the browning of white adipose tissue (17), alleviate hepatosteatosis, improve cognitive functions, and stimulate osteoclastogenesis and bone resorption (18–21). Thus, it would be vital to reveal possible alternations of myokine gene expressions in the skeletal muscle during NAFLD and NASH, which may provide more insights for mimicking an exercise in combating liver diseases.

In the present study, we investigated the transcriptome changes of quadriceps (QU) muscle, one of the largest muscles with both oxidative and glycolytic fibers, in mice of NAFLD and NASH model using RNA sequencing. Through bioinformatic analysis, we discovered the similarity and heterogeneity of altered gene programs in the QU muscle of NAFLD and NASH mice and determined the core network of these gene programs. We also investigated the altered myokines to understand the possible contribution of skeletal muscle on the progression of liver disease *via* muscle–liver crosstalk. Overall, the comprehensive transcriptomic analysis would provide a molecular basis for the understanding of NAFLD and NASH in the aspect of the skeletal muscle.

## Methods

### Animal Experiments

Male C57BL/6J mice were purchased from Shanghai Research Center for Model Organisms, and all experimental procedures and animal care were following the guidelines of the Ethics Committee of Animal Experiments of East China Normal University. The mice were maintained under specific-pathogen-free conditions at 22°C as the standard housing temperature and under a 12-h light/dark cycle with free access to food and water. The NAFLD group of mice was fed with high-sucrose and high-fat (HSHF) diet with 40 kcal% fat, 20 kcal% fructose, and 2% cholesterol (D09100310, Research Diets) for 12 weeks, and the NASH group of mice was fed with HSHF diet for 28 weeks, while the normal group was fed with control chow diet (Medicience Ltd., MD17111). The mice were anesthetized with sodium pentobarbital (Nembutal, 80 mg/kg, ip) and sacrificed. Samples, such as the liver and quadriceps muscles, were harvested for further analysis.

### Histological Analysis

Formalin-fixed, paraffin-embedded mouse liver sections were stained. Liver fibrosis was assessed by Sirius red (Polysciences, catalog 24901) staining as per the manufacturer’s instructions. Frozen sections from NAFLD and NASH mice were stained with 0.5% oil red O staining reagent for 20 min and were counterstained with hematoxylin for 5 min, and H&E staining was performed for the quantitative analysis of lipid accumulation in the liver. The different histological features of NAFLD and NASH were assessed using the NASH Clinical Research Network Scoring System as described previously (22).

### RNA Extraction, Transcriptome Analysis, and qPCR Analysis

Quadriceps muscles from NAFLD, CON1, NASH, and CON2 groups of mice were collected and frozen for subsequent RNA extraction. We have randomly selected 3 quadriceps muscle samples from each group of mice for RNA sequencing and 6 samples for qPCR confirmation. Total RNA was extracted from the liver or quadriceps muscle tissues using RNAiso Plus (Takara, 9108), following the manufacturer’s instructions, and the concentration was determined *via* NanoDrop Microvolume Spectrophotometers and Fluorometer (Thermo Fisher Scientific).

The RNA quality was checked using Bio-analyzer instrument (Agilent, USA), quantified with ND-2000 (NanoDrop Technologies), and then subjected to the construct cDNA library (CloudSeq Biotech Inc., Shanghai, China). Briefly, Ploy A mRNA was enriched using Oligo dT magnetic beads from 2 µg total RNA and broken up to 200 bp for each replicate. The double-strand cDNA was synthesized and purified for end repair, poly A addition, and adapter ligation. Then, the products enriched with PCR amplification generated cDNA libraries that were subsequently sequenced on Illumina HiSeq™ 3000 platform. The RNA-seq raw reads generated by Illumina sequencer were performed by trimming of adaptors and removal of low-quality reads using Skewer (v0.2.2). FastQC (v0.11.5) was used to check the quality of the pre-treated data which were mapped to GRCm38 using STAR (2.5.3a). The transcripts were assembled using StringTie (v1.3.1c), and the differential gene transcript expression between the early- and late-phase groups was analyzed with DESeq2 (v1.16.1). The differential threshold value was *P*-value ≤0.05 and fold-change ≥1.5.

For the qPCR analysis, 1 μg of total RNA was reverse-transcribed as cDNA by PrimeScript™RT Master Mix (Takara, RR036A). The qPCR analysis was performed on quantitative real-time PCR system (Roche, LightCycler 480) with SYBR Green Master Mix (Thermo Fisher Scientific, 4309155). Internal control using Gapdh gene for data analysis and cycle threshold (Ct) values was calculated using the 2^-ΔΔCt^ method. The primer sequences used in this study are shown in **Supplementary Table S1**.

### Western Blots

Protein extraction was performed with RIPA buffer containing 50 mM Tris (pH 7.4), 150 mM NaCl, 1% Triton X-100, 1% sodium deoxycholate, 0.1% SDS, sodium orthovanadate, sodium fluoride, EDTA, leupeptin, supplemented with 1 mM PMSF, 10 mM DTT, and 10 μM protein kinase inhibitor. The protein concentrations were quantified using a BCA protein quantification kit (Beyotime), and the samples from the SDS-PAGE gels were transferred to a membrane of nitrocellulose by electrophoretic transfer. The membranes were blocked with 5% skimmed milk for 1 h at room temperature and then incubated with anti-MSTN (Santa Cruz, sc-393335) and anti-GAPDH (Aksomics Biotechnology, KC-5G4) antibodies. The membranes were washed with Tris-buffered saline with Tween three times and incubated with IRDye secondary antibodies (926-68071 and 926-322210) from LI-COR Biosciences (NE, USA). The protein bands were detected by Odyssey^®^ CLX imaging system (LI-COR Biosciences, NE, USA).

### Gene Ontology and Kyoto Encyclopedia of Genes and Genomes Functional Enrichment Analyses of Differentially Expressed Genes

Database for Annotation, Visualization, and Integrated Discovery Functional Annotation Tool (23, 24) was used to perform Gene Ontology (GO) terms, including biological process (BP), cell component, and molecular function, as well as Kyoto Encyclopedia of Genes and Genomes (KEGG) pathway enrichment analysis. The top altered GO terms and KEGG pathways are shown in **Supplementary Table S2**.

### PPI Network and Module Analysis

The protein–protein interaction (PPI) network was built by using the Search Tool for the Retrieval of Interacting Genes (http://string.embl.de/) (25). The uploaded genes were clustered into networks to detect significant functional modules. Cytoscape (26) was used to subsequently visualize the PPI network and to calculate the properties of the network based on the interaction pair information.

### Statistical Analysis

All the data are represented as mean ± SEM. The data analysis was performed using GraphPad Prism7. The data normality test was examined with Shapiro–Wilk normality test. Two-tailed *t*-test analysis was performed to identify differences between two groups. *P*-value <0.05 was considered statistically significant.

## Results

### The Establishment of NAFLD and NASH Animal Models

**Figure 1:**
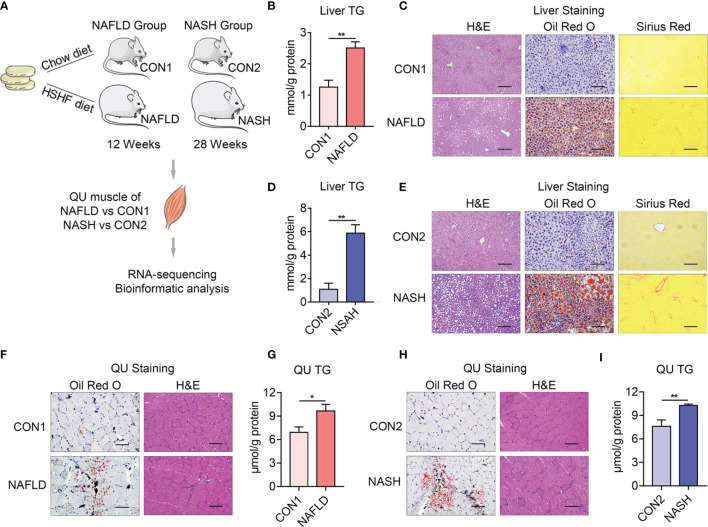
Establishment of animal models of nonalcoholic fatty liver disease (NAFLD) and nonalcoholic steatohepatitis (NASH). **(A)** Schematic diagram of the animal experiment design. Six 8-week-old, male C57BL/6J mice were fed with a high-sucrose and high-fat diet (40 kcal% fat, 20 kcal% fructose, and 2% cholesterol) for 12 weeks (as NAFLD group) or 28 weeks (as NASH group), while an age-matched control group was fed with chow diet for 12 weeks (as CON1 group) or 28 weeks (as CON2 group) as previously reported (28). Three quadriceps (QU) muscle samples from each group of mice for RNA sequencing and 6 samples for qPCR confirmation. **(B–I)** Comparison of the parameters of liver and skeletal muscle from the NAFLD and NASH groups of mice and their controls. **(B, C)** Hepatic triglyceride (TG) levels **(B)** and representative H&E, oil red O, and Sirius red staining of liver **(C)** from NAFLD and CON1 groups of mice. **(D, E)** Hepatic TG levels **(D)** and representative H&E, oil red O, and Sirius red staining of liver **(E)** from the NASH and CON2 groups of mice. **(F, G)** Representative oil red O and H&E staining **(F)**. TG levels of QU muscle **(G)** from the NAFLD and CON1 groups of mice. **(H, I)** Representative oil red O and H&E staining **(H)**. TG levels of QU muscle **(I)** from the NASH and CON2 groups of mice. Data are presented as mean ± SEM. *P < 0.05, **P < 0.01 compared to the control group. The scale bar represents 100 μm. *n* = 6 per group.

It has been reported that the development of NAFLD and NASH is accompanied with skeletal muscle dysfunctions (27). To understand the detailed molecular changes of skeletal muscles in NAFLD and NASH, we established NAFLD and NASH mice models by feeding mice with HSHF diet (40 kcal% fat, 20 kcal% fructose, and 2% cholesterol) for 12 weeks (as the NAFLD group) or 28 weeks (as the NASH group) as previously reported (28, 29). Their control group of mice was fed with chow diet (Medicience Ltd., MD17111) for 12 weeks (CON1) or 28 weeks (CON2) separately (**Figure 1A**). A detailed analysis on liver morphology revealed increased lipid accumulation in the livers of both the NAFLD and NASH groups of mice, in accordance with increased triglyceride contents, and a strong fibrotic area in the livers of the NASH group as shown by hematoxylin–eosin (H&E), oil red O, and Sirius red staining (**Figures 1B–E**).

**Figure 2:**
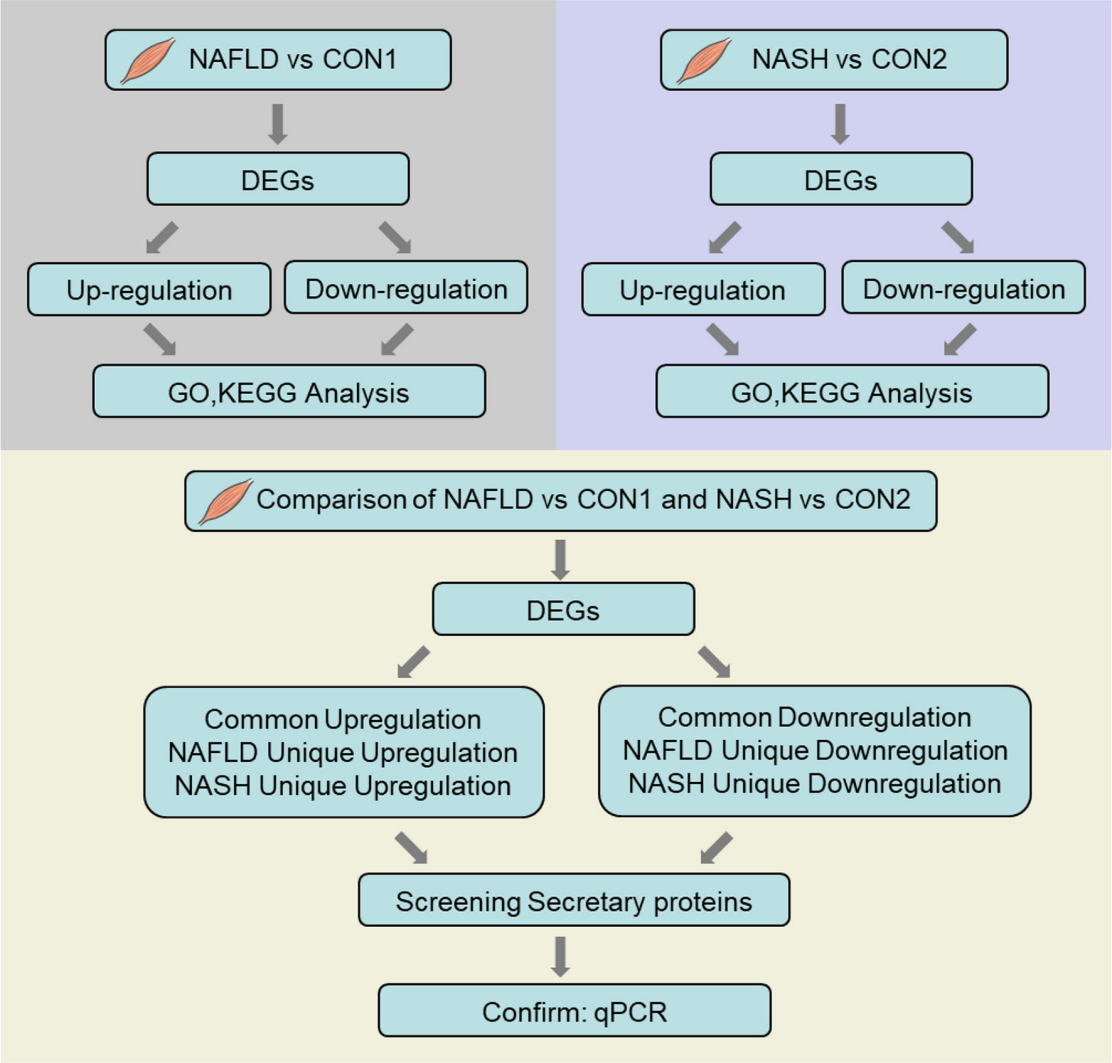
Flow chart of RNA sequencing data processing. The illustrated figure shows the bioinformatic analytic processes of common and differential changes in gene transcriptome among groups of nonalcoholic fatty liver disease (NAFLD) *vs*. CON1 and nonalcoholic steatohepatitis (NASH) *vs*. CON2 and comparison of differentially expressed genes between NAFLD *vs*. CON1 and NASH *vs*. CON2 with Gene Ontology and Kyoto Encyclopedia of Genes and Genomes pathway functional enrichment analysis.

The QU muscle is one of the largest skeletal muscles mixed with oxidative and glycolytic muscle fibers. Histological analysis and oil red O staining showed increased lipid deposition, which was confirmed by increased triglyceride contents in the QU muscle in the NAFLD and NASH groups of mice (**Figures 1F–I**). To comprehensively understand the molecular signature of the skeletal muscle in NAFLD and NASH, compared to their age-matched controls, we further took QU muscle samples for RNA sequencing (**Figure 2**).

### Induction of Secretome and Reduction of Muscle Functionality in QU Muscle in the NAFLD Group of Mice

**Figure 3:**
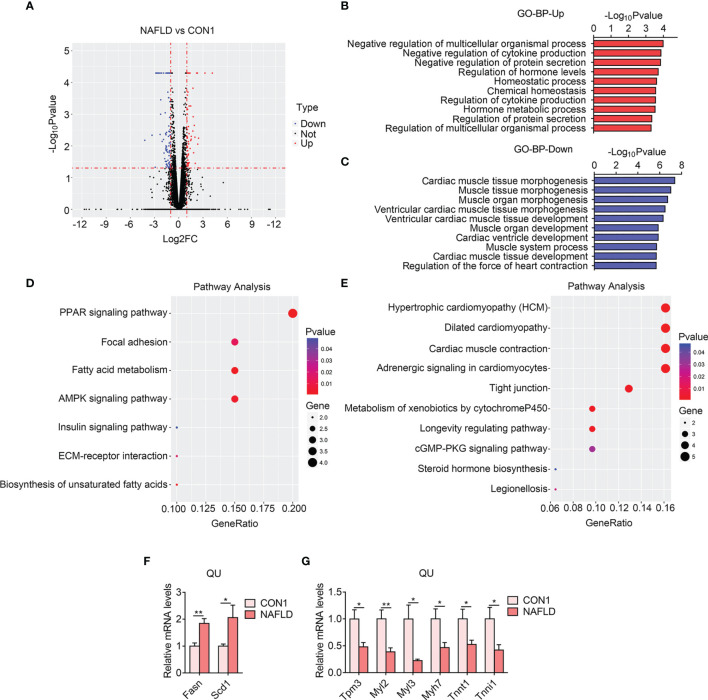
Transcriptome analysis of quadriceps (QU) muscle in the nonalcoholic fatty liver disease (NAFLD) *vs*. CON1 groups of mice. **(A)** Volcano plot showing differentially expressed genes (up- and down-regulated) from QU muscles in the NAFLD group compared with the CON1 group. The red dots represent the upregulated genes, and the blue dots represent the downregulated genes. **(B, C)** Gene Ontology analysis of the biological process of upregulated **(B)** and downregulated **(C)** differential genes from QU muscles in the NAFLD group compared to the CON1 group. **(D, E)** Kyoto Encyclopedia of Genes and Genomes analysis assessing the pathways associated with the upregulated **(D)** and downregulated € gene sets from QU muscles in the NAFLD group compared to the CON1 group. **(F, G)** Relative mRNA expression levels of up-regulated **(F)** and down-regulated **(G)** top altered genes in the NAFLD group of mice compared with CON1 group of mice (n=6). Data are presented as mean ± SEM. *P < 0.05, **P < 0.01 compared to the control group.

*Via* RNA sequencing, we firstly identified 140 (54 upregulated and 86 downregulated) differentially expressed genes in the NAFLD group of mice as shown by a volcano plot (**Figure 3A**). The GO analysis of BP revealed that cytokine production and secretion, protein secretion, and regulation of hormone level were increased (**Figure 3B**), suggesting that a crosstalk between the muscle and the metabolic organs happened in NAFLD, consistent with a previous finding that blocking cytokines, such as IL6, in NAFLD mice could alleviate insulin resistance in the skeletal muscle (30, 31). In addition, the KEGG pathway analysis demonstrated that the PPAR signaling pathway, fatty acid metabolism, and AMPK signaling pathway were upregulated (**Figure 3D**), which were consistent with previous reports claiming that these pathways play critical roles in lipid and glucose metabolism on NAFLD (32). Besides these, muscle morphogenesis, muscle development, muscle contraction, and hypertrophic pathways were reduced in the QU muscle from the NAFLD group compared with the control group, as shown by GO and KEGG analysis (**Figures 3C, E**), suggesting the decline of muscle functionality. Interestingly, these declined genes are also shared by the cardiac system. Indeed NAFLD patients are reported to be associated with inflammation-related atrial and ventricular myopathy, which is the predictor of coronary heart disease (33, 34). Notably, we validated the changes of the top altered gene using qPCR and found that *Fasn* and *Scd1* were increased and that *Tpm3*, *Myl2*, *Myl3*, *Myh7*, *Tnnt1*, and *Tnni1* were reduced in the NAFLD group compared with the controls (**Figures 3F, G** and **Supplementary Table S2**). Overall, these data suggested that NAFLD is associated with increased lipid metabolism and inflammation, while it suppressed muscle functionality in the skeletal muscle.

### Increased Lipid and Carbohydrate Metabolism and Reduced Muscle Functionality in the QU Muscle of the NASH Group of Mice

**Figure 4:**
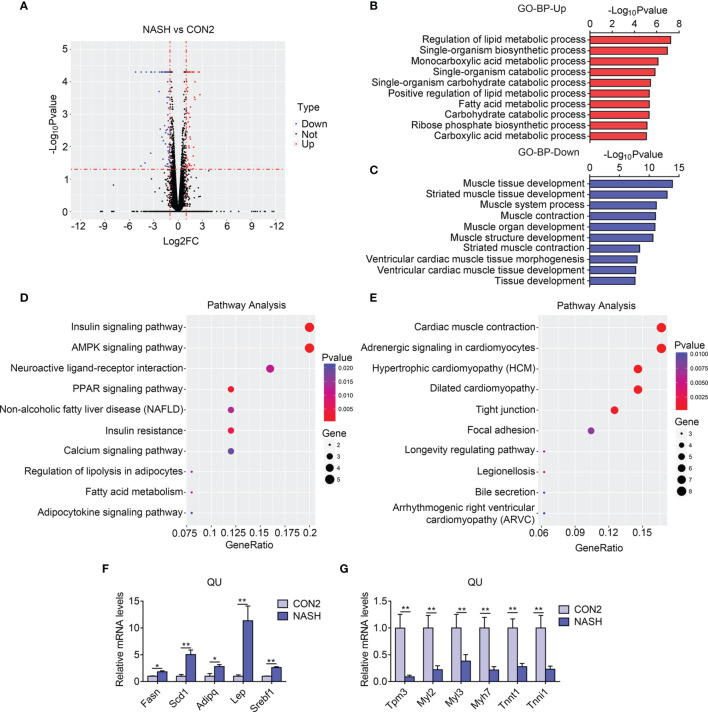
Transcriptome analysis of quadriceps (QU) muscle in the nonalcoholic steatohepatitis (NASH) *vs*. CON2 groups of mice. **(A)** Volcano plot showing differentially expressed genes (up- and downregulated) from QU muscles in the NASH group compared with the CON2 group. The red dots represent the upregulated genes, and the blue dots represent the downregulated genes. **(B, C)** Gene Ontology analysis of the biological process of upregulated **(B)** and downregulated **(C)** differential genes from QU muscles in the NASH group compared to the CON2 group. **(D, E)** Kyoto Encyclopedia of Genes and Genomes analysis assessing the pathways associated with the upregulated **(D)** and downregulated **(E)** gene sets from QU muscles in the NASH group compared to the CON2 group. **(F, G)** Relative mRNA expression levels of up-regulated **(F)** and down-regulated **(G)** top altered genes in the NASH group of mice compared with CON2 group of mice (n=6). Data are presented as mean ± SEM. *P < 0.05, **P < 0.01 compared to the control group.

Similarly, *via* RNA sequencing, data analysis identified 141 differentially expressed genes, including 53 upregulated and 88 downregulated genes, in NASH group of mice as shown in a volcano plot (**Figure 4A**). The GO enrichment analysis of biological process suggested that the regulation of lipid metabolic process and carbohydrate catabolic process was enriched to be upregulated (**Figure 4B**). Consistently, the KEGG pathway analysis revealed that the PPAR signaling pathway, fatty acid metabolism, and AMPK signaling pathway were dramatically enriched in upregulated genes in the skeletal muscle of the NASH group of mice (**Figure 4D**). Besides this, the GO analysis revealed that muscle tissue morphology, muscle development, and muscle contraction were downregulated in the NASH group muscle, similar to NAFLD (**Figure 4C**), which was consistent with the KEGG analysis showing that muscle contraction, adrenergic signaling, and hypertrophic genes were reduced (**Figure 4E**). Besides this, we confirmed the top altered genes by qPCR and found that *Fasn*, *Scd1*, *Adipoq*, *Lep*, and *Srebf1* were largely increased, while *Tpm3*, *Myl2*, *Myl3*, *Myh7*, *Tnnt1*, and *Tnni1* were sharply reduced in the NASH group in comparison with the control group (**Figures 4F, G** and **Supplementary Table S2**). Overall, these results suggested an increase in lipid and carbohydrate metabolism and a reduction of muscle development and functions in the skeletal muscle in NASH.

### Comparative Analysis Revealed That NAFLD and NASH Exposure Commonly Increases Fatty Acid Metabolism Pathways and Decreases Muscle Function

**Figure 5:**
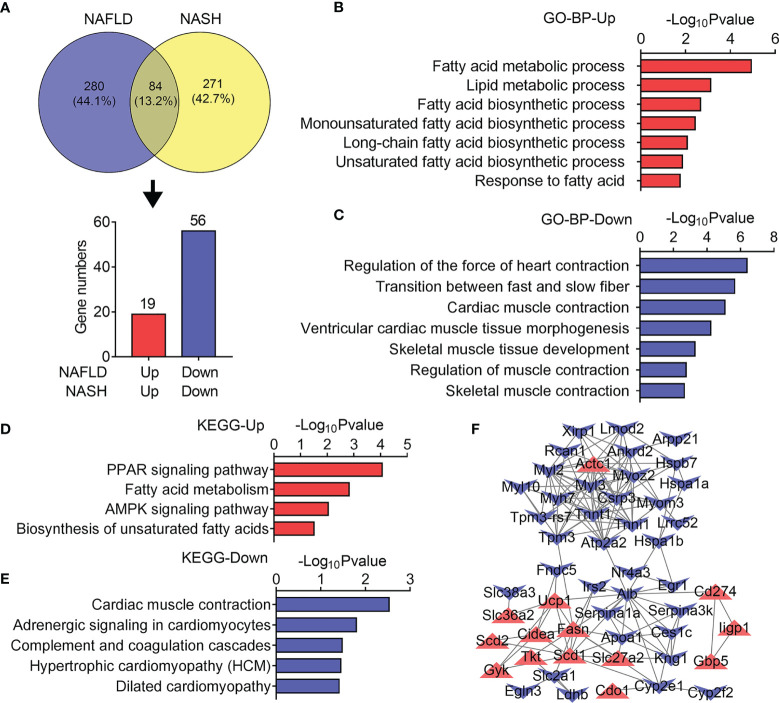
Common transcriptome analysis of quadriceps (QU) muscle between the nonalcoholic fatty liver disease (NAFLD) group and the nonalcoholic steatohepatitis (NASH) group of mice. **(A)** Venn diagram showing the differentially expressed genes of NAFLD and NASH mice compared with their controls. **(B, C)** Gene Ontology analysis of the biological process of upregulated **(B)** and downregulated **(C)** overlapping differential genes between the NAFLD and NASH groups of mice. **(D, E)** Kyoto Encyclopedia of Genes and Genomes analysis assessing the pathways associated with the upregulated **(D)** and downregulated **(E)** overlapping differential genes between the NAFLD and NASH groups of mice. **(F)** Protein–protein interaction network analysis of the overlapping differential genes between the NAFLD and NASH groups of mice.

We next set out to explore the common altered gene programs in both the NAFLD and NASH groups. Overlapping analysis identified 84 genes as displayed by a Venn diagram (**Figure 5A**). Among them, 19 upregulated and 56 downregulated genes were analyzed. In accordance with previous data, the GO and KEGG analysis showed that 19 upregulated genes were associated with fatty acid and lipid metabolic processes (**Figure 5B**) as well as the PPAR signaling pathway, fatty acid metabolism, and AMPK signaling pathway (**Figure 5D**). Besides this, 56 downregulated genes were related to muscle contraction and development as well as to muscle contraction, adrenergic signaling, and hypertrophic gene programs (**Figures 5C, E**). Furthermore, we examined protein–protein interactions for upregulated and downregulated genes with a PPI network (**Figure 5F**) and found that lipid synthetic genes like *Fasn*, *Scd1*, and *Scd2* showed dramatic increases, while muscle fiber markers including *Tnni1*, *Tnnt1*, *Myl3*, and *Myh7* showed sharp decreases. Overall, these results suggested increased lipid metabolism, majorly lipid synthetic genes, and reduced muscle developmental and functional genes that were commonly regulated genes in the skeletal muscle of both NALFD and NASH mice.

### Analysis of Unique Gene Programs in NAFLD and NASH Mice

**Figure 6:**
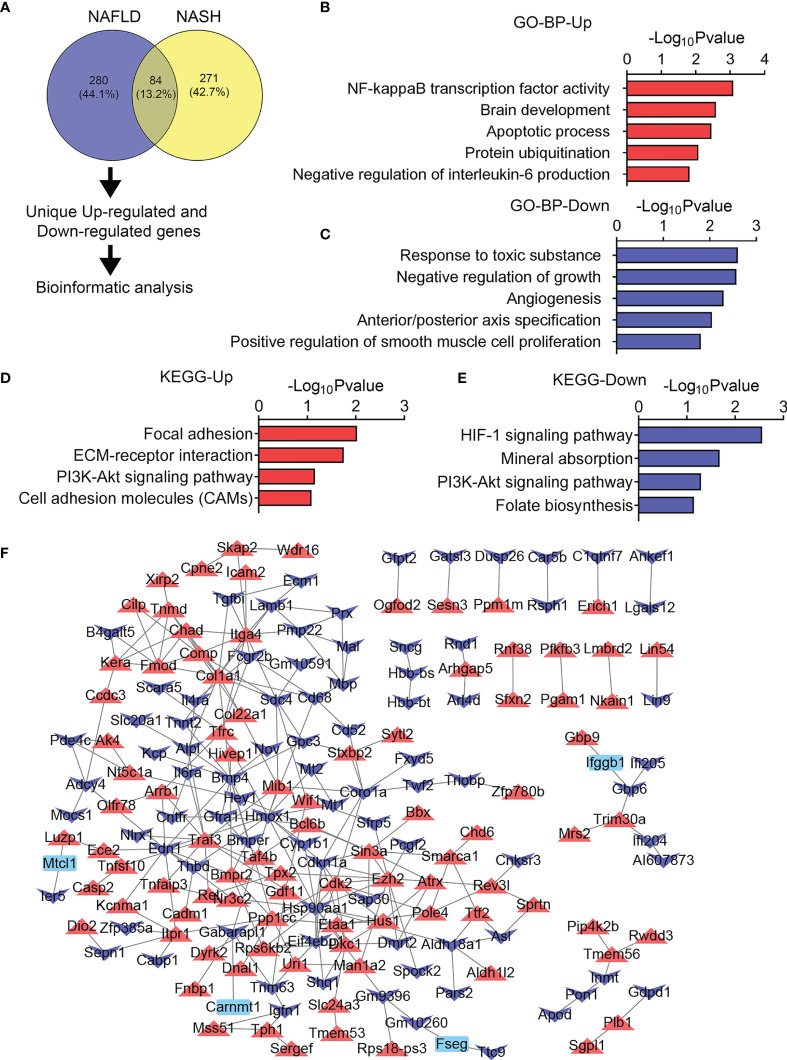
Unique transcriptome analysis of quadriceps (QU) muscle in the nonalcoholic fatty liver disease (NAFLD) group of mice. **(A)** Unique differential genes in the NAFLD group of mice. **(B, C)** Gene Ontology analysis of the biological process of upregulated **(B)** and downregulated **(C)** unique differential genes in the NAFLD group of mice. **(D, E)** Kyoto Encyclopedia of Genes and Genomes analysis assessing the pathways associated with upregulated **(D)** and downregulated **(E)** unique differential genes in the NAFLD group of mice. **(F)** Protein–protein interaction analysis of unique differential genes in the NAFLD group of mice.

Next, we investigated the heterogeneity in gene expression patterns in the skeletal muscle of NAFLD and NASH mice. For 280 DEGs uniquely changed in the skeletal muscle of the NAFLD group of mice (**Figure 6A**), the GO and KEGG analysis reveal that NF-kB transcription factor activity and apoptotic processes were uniquely upregulated (**Figures 6B, D**), suggesting inflammatory and apoptotic events that happen in the early stage of liver diseases as reported previously (35); meanwhile, chronic aerobic exercise could ameliorate it (36). Besides this, we found that response to toxic substance, regulation of growth, angiogenesis, axis specification as well as HIF-1 signaling pathway, mineral absorption, PI3K-Akt signaling pathway were enriched in NAFLD uniquely downregulated genes (**Figures 6C, E**), suggesting that the skeletal muscle may exhibit a disturbance in response to a toxic substance and muscle growth. Furthermore, the PPI network diagram revealed that NF-kB and transforming growth factor-β signaling pathway, including *Tab2*, *Map3k7*, *Traf3*, *Tgfβ3*, and *Tgfβ2*, were highly induced (**Figure 6F**).

**Figure 7:**
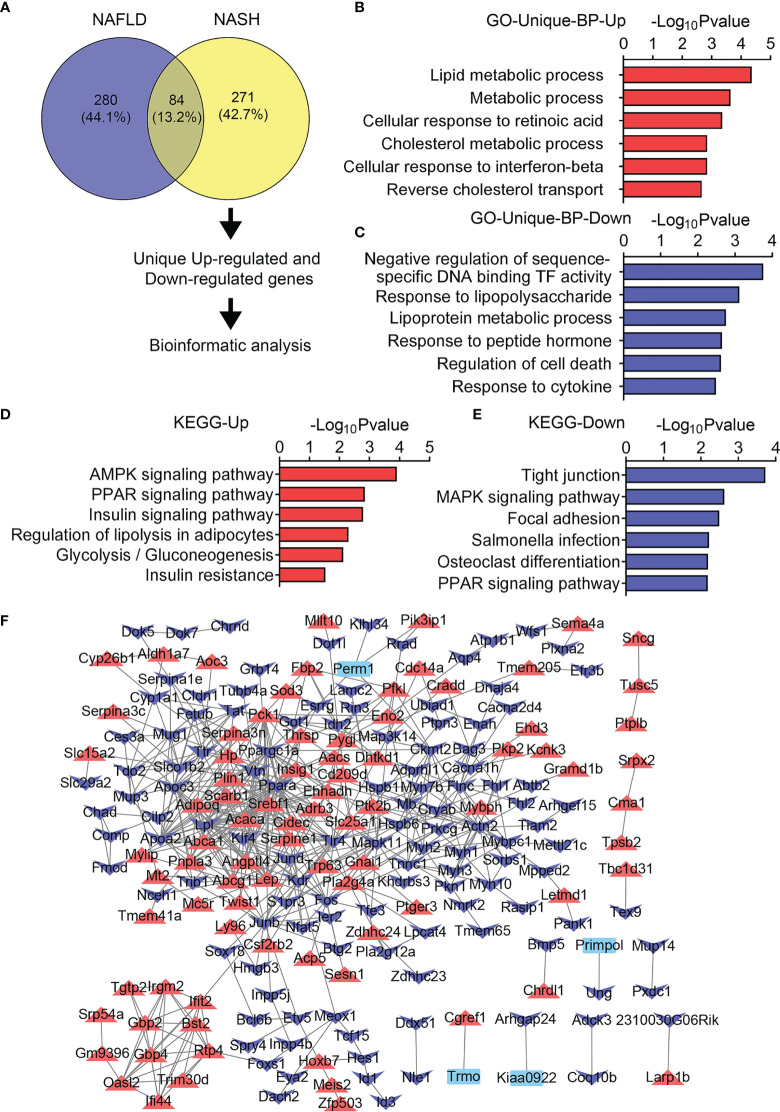
Unique transcriptome analysis of quadriceps (QU) muscle in the nonalcoholic steatohepatitis (NASH) group of mice. **(A)** Unique differential genes in the NASH group of mice. **(B, C)** Gene Ontology analysis of the biological process of upregulated **(B)** and downregulated **(C)** unique differential genes in the NASH group of mice. **(D, E)** Kyoto Encyclopedia of Genes and Genomes analysis assessing the pathways associated with upregulated **(D)** and downregulated **(E)** unique differential genes in the NASH group of mice. **(F)** Protein–protein interaction analysis of unique differential genes in the NASH group of mice.

Moreover, 271 DEGs were uniquely changed in the skeletal muscle in the NASH group of mice (**Figure 7A**). Notably, lipid metabolic process as well as AMPK signaling pathway, PPAR signaling pathway, and insulin signaling pathway were uniquely upregulated in NASH mice as shown by the GO and KEGG analysis (**Figures 7B, D**), suggesting enforced lipid deposition and enhanced lipid and glucose metabolic dysfunctions in the skeletal muscle of NASH than the NAFLD group, while lipoprotein metabolic process as well as tight junction, MAPK signaling pathway, and focal adhesion were uniquely downregulated in the muscle from the NASH group (**Figures 7C, E**). In addition, the PPI network analysis revealed increased levels of *Adipoq*, *Srebf1*, *Lep*, *Fabp4*, and *Cebpa*, which were associated with increased lipid deposition, in the NASH group of mice (**Figure 7F**).

### Identification of Myokines Commonly Altered in the Skeletal Muscle of NAFLD and NASH Groups of Mice

**Figure 8:**
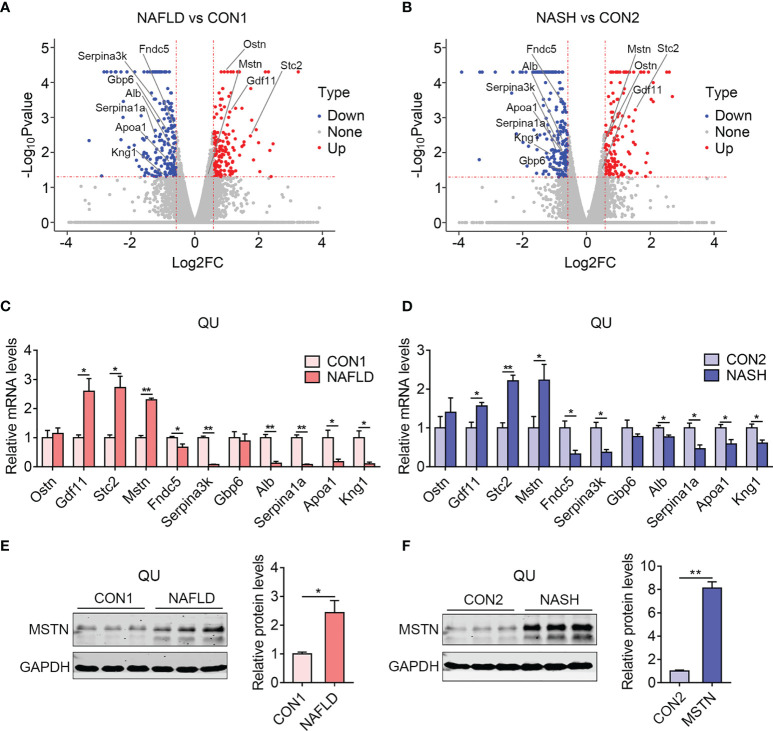
Identification of myokines commonly altered in the skeletal muscle of the nonalcoholic fatty liver disease (NAFLD) and nonalcoholic steatohepatitis (NASH) groups of mice. **(A, B)** Volcano diagram showing overlapping myokines altered in the NAFLD **(A)** and NASH groups of mice **(B)** compared with their controls. **(C, D)** Relative mRNA expression levels of myokines in the NAFLD **(C)** and NASH groups of mice **(D)** compared with their controls (*n* = 6). **(E, F)** Representative protein levels of MSTN in the NAFLD **(E)** and NASH groups of mice **(F)** compared with their controls (*n* = 3). Data are presented as mean ± SEM. *P < 0.05, **P < 0.01 compared to the control group.

Lastly, to understand the possible crosstalk between muscle and other metabolic organs, including liver, we analyzed commonly altered myokines in skeletal muscle from the NAFLD and NASH groups of mice compared to their controls. Notably, 4 upregulated myokines (*Mstn*, *Stc2*, *Gdf11*, and *Ostn*) and 8 downregulated myokines (*Serpina3k*, *Serpina1a*, *Kng1*, *Gbp6*, *Apoa1*, *Alb*, *Fndc5*, and *Irisin*) were shown in volcano plots (**Figures 8A, B** and **Supplementary Table S3**). The changes of majority of these myokines were confirmed by qPCR, while *Ostn* and *Gbp6* in the NAFLD and NASH groups showed marginal changes without significance (**Figures 8C, D**). Besides this, we validated the protein levels of MSTN, a classic atrophic marker gene that promotes muscle wasting. Notably, the protein expression of MSTN was induced in the quadriceps muscle of both the NAFLD and NASH groups compared with their controls (**Figures 8E, F**).

Notably, among these genes, the increased levels of *Mstn*, *Gdf11*, and *Stc2* have been reported to be involved in muscle wasting or muscle dysfunction (37–39). Besides this, *Fndn5/Irisin* and *Apoa1*, which have been shown to promote muscle hypertrophy and the systematic improvement of lipid and glucose metabolism (40–42), were largely declined in the muscle of NAFLD and NASH mice, overall suggesting the possible contribution of myokines on the systematic and pathological performance during NAFLD and NASH. In addition, other genes, including Serpina3k and Serpina1a, have not been well understood as myokines and might be involved in muscle functionality and whole-body metabolism during NAFLD and NASH.

In addition, we investigated the uniquely changed myokines in the NASH group and found that *Lep*, *Ly96*, *Hp*, *Adipoq*, and *Serpina3n* were uniquely upregulated, while *Chad*, *Cilp2*, *Wif1*, *Fetub*, *Comp*, and *Mup14* were uniquely downregulated in the NASH group compared with the CON2 groups (**Supplementary Table S3**), which were potentially affected by NASH progression.

## Discussion

NAFLD and NASH are the most prevalent chronic liver diseases accompanied with skeletal muscle dysfunctions. Emerging evidences suggest that reduced skeletal muscle mass and strength contribute to the progression of NAFLD or NASH (27, 43, 44). However, the molecular alterations are not well understood in the skeletal muscle at different stages of NAFLD or NASH. In this study, we identified the DEGs in QU muscles with NAFLD and NASH in comparison to normal controls using RNA sequencing, followed with comprehensive and comparative analysis, as well as identified myokines that are possibly involved in tissue crosstalk in the pathological states. Overall, *via* GO analysis, we found that lipid metabolism and inflammatory genes were dramatically increased, while skeletal muscle development, functionality, and contraction were reduced in QU muscle from NAFLD and NASH mice compared with their controls. Consistently, the KEGG pathway functional enrichment analysis has shown that classic lipid and glucose signaling pathways, including PPAR, AMPK, and insulin receptors, were enriched, suggesting that metabolic dysfunction may be the primary cause for muscle dysfunction. In addition, we identified key myokines altered in NAFLD and NASH mice and confirmed with qPCR, which might provide potential therapeutic targets for the treatment of NAFLD and NASH in the aspect of improving muscle functionality.

Our analysis revealed perturbations in the lipid metabolism of skeletal muscle in NAFLD and NASH. Indeed it has been reported that a high-fat diet results in increased lipid metabolism in the skeletal muscle at both transcriptional and metabolite levels, including accumulation of glycerophospholipid and acyl carnitine species (45). In addition, short-term high-fat (45 kcal%) palm oil diet feeding leads to similar effects on lipid metabolism in rodent skeletal muscle (46). In humans, it has been demonstrated that genes controlling fatty acid metabolism were increased in human skeletal muscles from patients with metabolic diseases (47, 48). Notably, we found that the signaling of PPARα, a ligand-activated nuclear receptor governing fatty acid metabolism in the muscle and liver, was highlighted to be enriched in the muscle of NAFLD and NASH. Indeed it has been shown that PPARα activation enhances lipid turnover and alleviates the development of dietary steatohepatitis, while PPARα^-/-^ mice exacerbated steatohepatitis (49). However, it has to be noted that the expression of PPARα is enhanced in the gastrocnemius muscle of obese mice (50), and PPARα is reported to be activated in skeletal muscle from type 2 diabetes mellitus patients, suggesting a possible compensatory mechanism (51). Considering that PPARα agonists fibrates, such as fenofibrates, have been widely used clinically to treat hyperlipidemia and, in some cases, NAFLD and NASH, it would be interestingly to further investigate whether fibrates also exert effects in the skeletal muscle.

Besides this, previous reports have shown that increased intramyocellular droplet storage results in the accumulation and dysregulation of detrimental lipid intermediates, such as diacylglycerols and ceramides, which leads to insulin resistance by the activation of PKC and contributes to the development of muscle loss in obese human subjects (23, 24), suggesting that lipid dysfunction in muscle also affects insulin resistance and muscle atrophy in patients with NAFLD and NASH [8]. Indeed we found that the insulin receptor signaling was enriched in the QU muscle of NAFLD and NASH. NAFLD and NASH are commonly seen in patients with type 2 diabetes (T2D), and over 50% of T2D patients are diagnosed with liver steatosis (52). The skeletal muscle is the primary organ responsible for glucose disposal by means of insulin-mediated mechanism and is of great importance for sustaining glucose homeostasis and insulin resistance. In other words, insulin resistance in the skeletal muscle is a central pathogenic factor for the development of metabolic diseases such as NAFLD, NASH, and T2D (1, 53, 54). Thus, our data suggested that lipid overload and insulin resistance are the key phenotypes of skeletal muscle in NAFLD and NASH.

Muscle wasting is a risk factor for the development of NAFLD and NASH (55). Interestingly, in our data, skeletal muscle or cardiac muscle, organ, structure development, and contraction, which are associated GO terms on BPs, were significantly enriched in downregulated genes both in NAFLD and NASH mice compared with control littermates. In addition, the pathway analysis showed that cardiac muscle contraction, hypertrophic cardiomyopathy, and adrenergic signaling in cardiomyocyte signaling pathways were highly significantly enriched in downregulated gene sets, suggesting that the cardiovascular system is also severely affected in NAFLD and NASH, which was consistent with clinical reports (33, 34).

Notably, we identified several altered myokines in the skeletal muscle during NAFLD and NASH. Among them, *Fndc5/Irisin*, which is well known to promote the browning of white adipose tissue, enhances muscle hypertrophy and alleviates obesity and hepatic steatosis, was significantly decreased in both NAFLD and NASH groups. *Apoa1*, a major component of high-density lipoprotein known to improve glucose clearance and insulin sensitivity, was reduced in the NAFLD and NASH groups (40, 41). Besides this, *Mstn* and *Gdf11*, two key muscle atrophy markers (37–39), as well as *Stc2*, a secreted glycoprotein highly expressed in the skeletal muscle and heart as a potent growth inhibitor for reduced skeletal muscle mass in rodents and human, were increased in the NAFLD and NASH groups (56, 57), yet it would be more convincing to confirm these myokine changes *via* ELISA analysis. In addition, more myokines were not reported to be associated with muscle functionality or metabolic diseases, which need further investigations.

## Data Availability Statement

The original contributions presented in the study are publicly available. This data can be found here: NCBI, PRJNA798207, https://www.ncbi.nlm.nih.gov/search/all/?term=PRJNA798207

## Ethics Statement

The animal study was reviewed and approved by the Ethics Committee of Animal Experiments of East China Normal University.

## Author Contributions

JZ, XM, and LX devised and supervised the project. MG performed bioinformatic analysis. LX constructed the mouse models and harvested the samples. MG, JY, JZ, and SZ performed biochemical and molecular experiments. DW, CL, GL, JW, YG, and CX participated in experiments and discussions. XM wrote the manuscript. LX and JZ edited and revised the manuscript. All authors contributed to the article and approved the submitted version.

## Funding

This project is supported by funds from the National Key Research and Development Program of China (2019YFA0904500), the Science and Technology Commission of Shanghai Municipality (21140904300), the National Natural Science Foundation of China (32022034, 31770840, 32071148, and 31800989), the Fundamental Research Funds for the Central Universities, ECNU public platform for Innovation (011), and the instruments sharing platform of School of Life Sciences.

## Conflict of Interest

The authors declare that the research was conducted in the absence of any commercial or financial relationships that could be construed as a potential conflict of interest.

The reviewer BZ declared a shared affiliation with several of the authors (YG and CX) to the handling editor at the time of review.

## Publisher’s Note

All claims expressed in this article are solely those of the authors and do not necessarily represent those of their affiliated organizations, or those of the publisher, the editors and the reviewers. Any product that may be evaluated in this article, or claim that may be made by its manufacturer, is not guaranteed or endorsed by the publisher.
