# Supplementary material for: Comprehensive Transcriptome Profiling of NAFLD- and NASH-Induced Skeletal Muscle Dysfunction
Source: Front Endocrinol (Lausanne). 2022 Feb 21;13:851520. doi: 10.3389/fendo.2022.851520 (PMC8899658; doi:10.3389/fendo.2022.851520)
Supplement: Supplementary file 1 [file Table_1.doc]

**Supplementary Table S1. qPCR primers used in the study.**

| Gene name | Primer sequence (5′→3′) | |
| --- | --- | --- |
| mOstn | Forward | CTGGAGATTGGCAAGTACACAC |
|  | Reverse | CGCAGGAGCTTAGCCGAAA |
| mGdf11 | Forward | CCGGCGTCACATCCGTATC |
|  | Reverse | ACTTGCTTGAAGTCGATGCTC |
| mStc2 | Forward | CTGGGCCAGTTTGTGACCC |
|  | Reverse | ACAAGAGTTGTTCTCGAAACACT |
| mMstn | Forward | ACGAGTGGATGGTGCGCTGTGTGC |
|  | Reverse | TCATTCTGAACGCGCATGAAGCG |
| mFndc5 | Forward | ATGAAGGAGATGGGGAGGAA |
|  | Reverse | GCGGCAGAAGAGAGCTATAACA |
| mSerpina3k | Forward | AGAGGAGCTAAACCTGCCCAA |
|  | Reverse | ATACGGCCTTACGAATGCCAC |
| mGbp6 | Forward | GTTCCAGGAAGTAACAAAGGCT |
|  | Reverse | ATCCCTAGTCTATTCCCAGTGAC |
| mAlb | Forward | CAAGAGTGAGATCGCCCATCG |
|  | Reverse | TTACTTCCTGCACTAATTTGGCA |
| mSerpina1a | Forward | AGCATCTGGAGCAAACTCTC |
|  | Reverse | TCCAGAGATGGACAGTCTGG |
| mApoa1 | Forward | GCTCAAGAGCAACCCTACCTT |
|  | Reverse | GCTTTCTCGCCAAGTGTCTTC |
| mKng1 | Forward | TTAACCCTGGGGTAAAAAGTGGC |
|  | Reverse | TGGACTATCTGTCGATATGGCA |
| mFasn | Forward | GGAGGTGGTGATAGCCGGTAT |
|  | Reverse | GGAGGTGGTGATAGCCGGTAT |
| mScd1 | Forward | TTCTTGCGATACACTCTGGTGC |
|  | Reverse | CGGGATTGAATGTTCTTGTCGT |
| mAdipq | Forward | GAAGCCGCTTATGTGTATCGC |
|  | Reverse | GAATGGGTACATTGGGAACAGT |
| mLep | Forward | GAGACCCCTGTGTCGGTTC |
|  | Reverse | CTGCGTGTGTGAAATGTCATTG |
| mSrebf1 | Forward | GCAGCCACCATCTAGCCTG |
|  | Reverse | CAGCAGTGAGTCTGCCTTGAT |
| mTpm3 | Forward | AGAGGATGAACTAGCAACCATGC |
|  | Reverse | CTTCCGAATACTTGTCCAGCTC |
| mMyl2 | Forward | ATCGACAAGAATGACCTAAGGGA |
|  | Reverse | ATTTTTCACGTTCACTCGTCCT |
| mMyl3 | Forward | TGCCTCCAAGATTAAGATCGAGT |
|  | Reverse | CTCTGCCTGGGTAGGATTCTG |
| mMyh7 | Forward | GCCAACTATGCTGGAGCTGATGCCC |
|  | Reverse | GGTGCGTGGAGCGCAAGTTTGTCATAAG |
| mTnnt1 | Forward | TGGATCCACCAGCTGGAATCAGAA |
|  | Reverse | GCTGATGCGGTTGTAGAGCACATT |
| mTnni1 | Forward | TGAAGCCAAATGCCTCCACAACAC |
|  | Reverse | ACACCTTGTGCTTAGAGCCCAGTA |
| mGapdh | Forward | ACAACTTTGGCATTGTGGAA |
|  | Reverse | GATGCAGGGATGATGTTCTG |
